# Using Blue Mini-LEDs as a Light Source Designed a Miniaturized Optomechanical Device for the Detection of Direct Bilirubin

**DOI:** 10.1186/s11671-022-03750-z

**Published:** 2022-11-22

**Authors:** Zhi Ting Ye, Hsin-Ching Kuo, Shen Fu Tseng, Shu-Ru Chung, Shang-Xuan Tsou

**Affiliations:** 1 Department of Mechanical Engineering, Advanced Institute of Manufacturing with High-Tech Innovations, National Chung Cheng University, 168, University Rd., Min-Hsiung, Chia-Yi, 62102 Taiwan, ROC; 2 Ditmanson Medical Foundation Chia-Yi Christian Hospital, Chia-Yi, Taiwan, ROC; 3 Department of Materials Science and Engineering, National Formosa University, No. 64, Wunhua Rd., Huwei Township, 632 Yunlin County Taiwan, ROC

**Keywords:** Blue Mini-LEDs, Miniaturized optomechanical, Noninvasive, Direct bilirubin, Quantitative analysis, Coefficient of determination

## Abstract

This study developed a miniaturized optomechanical device (MOD) for the feasibility study of direct bilirubin in urine using high-collimation blue mini-light-emitting diodes (Mini-LEDs) as the light source. The constructed MOD used optical spectroscopy to analyze different concentrations of direct bilirubin using the absorbance spectrum to achieve a noninvasive method for detection. The experimental results showed that between the absorbance and different concentrations of direct bilirubin at the blue Mini-LEDs central wavelength (462 nm) was the optimum fitting wavelength; in the direct bilirubin concentration range from 0.855 to 17.1 μmol/L, the coefficient of determination (*R*^2^) was 0.9999, the limit of detection (LOD) of 0.171 μmol/L, and the limit of quantitation (LOQ) of 0.570 μmol/L. Therefore, we propose using blue Mini-LEDs as a light source to design a MOD to replace the invasive blood sampling method with a spectroscopic detection of direct bilirubin concentration corresponding to absorbance.

## Introduction

Bilirubin is a yellow pigment produced by the catabolism of hemoglobin in red blood cells and excreted from the body through urine and feces. Bilirubin is generally divided into three categories: direct bilirubin, indirect bilirubin, and total bilirubin (indirect plus direct bilirubin). Excess bilirubin causes the skin, eyes, and other tissues to turn yellowish-brown, a condition called jaundice [1–3]. Typically, full-term and premature babies have a 60% and 80% chance, respectively, of developing jaundice. As a result, bilirubin testing is a regular procedure performed on newborns [4, 5].

Jaundice occurring in newborns is generally classified as physiological or pathological jaundice. The majority of neonatal cases are physiological jaundice and do not have serious consequences. However, abnormally high bilirubin values can lead to neurotoxicity, and neurodevelopmental abnormalities, such as hearing loss, athetosis, and mental deficiencies [6–9]. Physiological jaundice in newborns typically appears on the second day of life, peaking on the fourth to fifth day, and then subsiding over a period of one to two weeks. The two main factors that account for pathological jaundice (cholestatic jaundice) are biliary atresia and neonatal hepatitis. In biliary atresia, early diagnosis and surgery are necessary to avoid the eventual need for a liver transplant. Neonatal jaundice lasting greater than two weeks, called delayed jaundice, should be investigated and further tests conducted to rule out pathological jaundice. When delayed jaundice is present in a newborn, the current recommendation of the American Academy of Pediatrics (AAP) is to draw blood for total and direct bilirubin testing to rule out the possibility of biliary atresia. The importance of indirect bilirubin detection in diagnosing jaundice is minimal; only direct bilirubin is filtered by the kidneys and excreted into the urine. In addition, indirect bilirubin is insoluble in water. Therefore, the values obtained by urinary assessment of bilirubin are dominated by analysis of direct bilirubin [10–12]. In Taiwan, there is a stool color card in the child health handbook for parents to compare the color of their infant’s stool for early screening of biliary atresia. However, parents and health care workers have concerns about making errors when comparing infants’ stools with the stool color cards. Therefore, it is hoped that developing a noninvasive urine bilirubin test will provide them with accurate and immediate results.

Most bilirubin assays used in clinical chemistry laboratories are based on diazotization and oxidase methods [13, 14]. In a typical automated diazo colorimetric method, bilirubin and diazo ions are reacted under strongly acidic conditions of pH 1.7–2.0 to form a reddish colored azo compound. The fluorescence intensity of the azo compound is measured at 600 nm using a photometer, and the bilirubin concentration is determined [15]. Saifee et al. proposed a method that mixes normal and ultra-filtered plasma samples with O-desmethylnaproxen (ODMN) to detect total bilirubin. Their experimental results showed that total bilirubin’s absorption was in the wavelengths between 450 and 560 nm when combined with ODMN. After combining with the jaundice samples, the best absorption wavelength was found to be 520 nm [16]. Ponhong et al. proposed a hybrid analytical system for the determination of direct bilirubin in urine samples, which uses n-octyl-β-d-thioglucoside (OTG) as the solubilizer for bilirubin; when it reacts with diazotized sulfanilic acid, OTG-azobilirubin is formed. Analysis of the linear relationship between the direct bilirubin concentration and the OTG-azobilirubin found the coefficient of determination (*R*^2^) was 0.994 in the concentration range of 0–1.0 mg/L, the detection limit was 4.7 μg/L, and the relative standard deviation value (RSD) was 1.9% [17]. Lano et al. proposed the analysis of total bilirubin concentration using a co-oxygenation saturation assay and Radiometer® ABL90; samples were centrifuged and analyzed for total bilirubin in plasma using diazo reagents. The mean deviation of the total bilirubin levels examined was − 1.0 µmol/L, which is more precise than spectrophotometric methods with a mean deviation of − 4.4 µmol/L [18].

Although the diazo assay for bilirubin is inexpensive and easy to automate, it is susceptible to hemolysis and lipid or albumin interference, causing a shift in the pH of the diazo reagent and unpredictable deviations in the determination of the bilirubin concentration Therefore, the current diazo detection method for bilirubin can only be considered a qualitative analysis, as its reliability has not been able to reach the high accuracy standards required for quantitative analysis [19–21].

Bilirubin oxidase (BOD) is a multicopper oxidase (MCO), which uses metal ions to catalyze the oxidation of bilirubin to biliverdin, and is used in the determination of bilirubin concentration. BOD exhibits high activity and stability at neutral pH and is widely used as a test reagent [22–25]. Rachna et al. developed a biosensor by immobilization of BOD on graphene oxide nanoparticles. The biosensor showed a linear dependence for bilirubin concentration from 0.01 to 600 μmol/L and retained about 80% of its initial activity when repeatedly utilized, about 100 times, over 180 days. Compared with the standard colorimetric technique, the biosensor’s correlation of coefficient (*r*) was 0.99 [26]. Zheng et al. fabricated a novel electrochemical sensor based on Au nanoparticles/tetrathiafulvalene-carboxylate functionalized reduced graphene oxide 0D-2D heterojunction for the detection of bilirubin in human blood. The electrochemical sensor had a linear response range of 2.66 to 83 μmol/L for bilirubin, with a detection limit of 0.74 μmol/L [27]. Rahman et al. used Mn(II) ions and conductive polymers for the development of a current-based bilirubin biosensor, which was coated with a polyethylenimine film of ascorbic acid oxidase; experimental data showed that the optimal linear range of bilirubin ranged from 0.1 to 50 μmol/L, with a detection limit of 40 ± 3.8 nmol/L, with 3.8 nmol/L showing good stability and a fast response less than 5 s [28].

Although the oxidase method for the detection of bilirubin is widely used, the threshold of the technique is high, there is interference by substances, such as albumin or hemoglobin, and the separation of enzymes must be restricted to aqueous solvents. Therefore, there is much room for improvement to achieve quantitative detection [29–32].

Quantum dots are semiconductor nanostructure materials that modulate the radiation wavelength by changing the particle size and have wide absorption and narrow emission. Because quantum dots obtain the same full width at half maximum (FWHM) at different and consistent emission wavelengths, they are increasingly used in fluorescence detection of biological samples [33–35]. Jayasree et al. proposed a new type of biosensor using quantum dots with an emitted wavelength of 405 nm that chemically reacts with Fe^3+^ to quench blue fluorescence. The study showed that fluorescence was regained by adding bilirubin. Linear regression analysis was done using the ratio of fluorescence quenching and bilirubin concentration. The *R*^2^ between the ratio of fluorescence quenching and bilirubin concentration was 0.99, and the limit of detection was 2.5 μmol/L. The data showed that the developed biosensor had good linearity of detection [36]. Zhao et al. reported a strategy to detect bilirubin using quantum dots modified with human serum albumin by cationic polymer polyethylenimine-induced aggregation, and then measuring the radioactive light intensity. This method was successfully applied to the quantitative determination of indirect bilirubin [37].

The fluorescence method detected purification bilirubin has a high linear correlation, but the detection method is dependent on chemical reactions. In addition, for biological samples with a complex composition, such as urine and blood, the anti-interference and linear correlation of fluorescence-based methods do not reach the expected level of accuracy [38–40]. Besides the methods above, the excess bilirubin in the blood will lead to the skin becoming yellow, and then, the method using diffuse reflectance spectroscopy to quantify bilirubin from skin reflectance spectra has been proposed [41]. There is also a noninvasive method by using a light beam incident on the nail and analyzing the reflected light through optical fibers based on diffuse reflection [42].

Compare LEDs with conventional light sources employed in analytical instruments, due to the narrow width of the LEDs emission spectrum, it is usually 30 nm which can improve the light utilization efficiency. The analytical application of LEDs is used in chromatography, absorption detection in capillaries, fluorescence spectrometry, microfluidic devices, photoacoustic spectroscopy [43].

The current noninvasive methods of bilirubin detection aim to quantify the total and direct bilirubin concentration. Because the blue wavelength of 462nm is very close to the maximum absorbance wavelength of direct bilirubin. Therefore, our study used blue Mini-LEDs as a light source to design a miniaturized optomechanical device (MOD) to replace invasive blood sampling methods. The device used the spectroscopic detection of the direct bilirubin concentration corresponding to the absorbance. The device offers the benefits of being noninvasive, fast, and providing a digitally quantitative analysis.

## Materials and Methods

### Absorbance Defined

When a parallel light beam is directed at an absorbing substance, after passing through a certain thickness of the substance it absorbs some of the light energy, and the intensity of the transmitted light decreases. The derivation process of the Beer–Lambert law is shown in Eqs. 1–2:

1 $${\text{Transmittance}} = T = \frac{{I_{1} }}{{I_{0} }}$$

2 $${\text{Absorbance}} = - \log_{10} T$$

where *T* is the transmittance, *A* is the absorbance value, *I*_0_ is the intensity of incident light, and *I*_1_ is the intensity of transmitted light.

According to the Beer–Lambert law, the absorbance value is proportional to the concentration and thickness of the substance, meaning that when the absorbing material is more concentrated or thicker, because of the greater absorbance of the material, the reduction in light intensity is more pronounced, as shown in Eq. 3:

3 $$A = \log_{10} \left( {\frac{{I_{0} }}{{I_{1} }}} \right) = \varepsilon \cdot \ell \cdot c$$

where *A* is the absorbance value, *ε* is the absorbance coefficient of the substance, $\ell$ is the thickness of the absorbing medium (cm), and *c* is the concentration of the absorbing substance (mg/dL).

Statistics commonly uses the *R*^2^ value to explain the correlation between the known data *X* (concentration) and the *Y* (absorbance) influenced by *X*. The *R*^2^ value is expressed as a value between 0 and 1; the larger the value or closer to 1, the better the relationship for the linear regression. The *R*^2^ value can be calculated using Eq. 4 [44]:

4 $$R^{2} = \sum (\widehat{{Y_{i} }} - \overline{Y})^{2} /\sum (Y_{i} - \hat{Y})^{2}$$

where $\widehat{{Y_{i} }}$ is the predicted value on the regression line, $\overline{Y}$ is the average of all *Y*_*i*_, $(\widehat{{Y_{i} }} - \overline{Y})^{2}$ is called the sum of squares for regression (SS_Reg_), and $(Y_{i} - \hat{Y})^{2}$ is the sum of squares for total (SS_Total_).

### Direct Bilirubin Sample

**Fig. 1:**
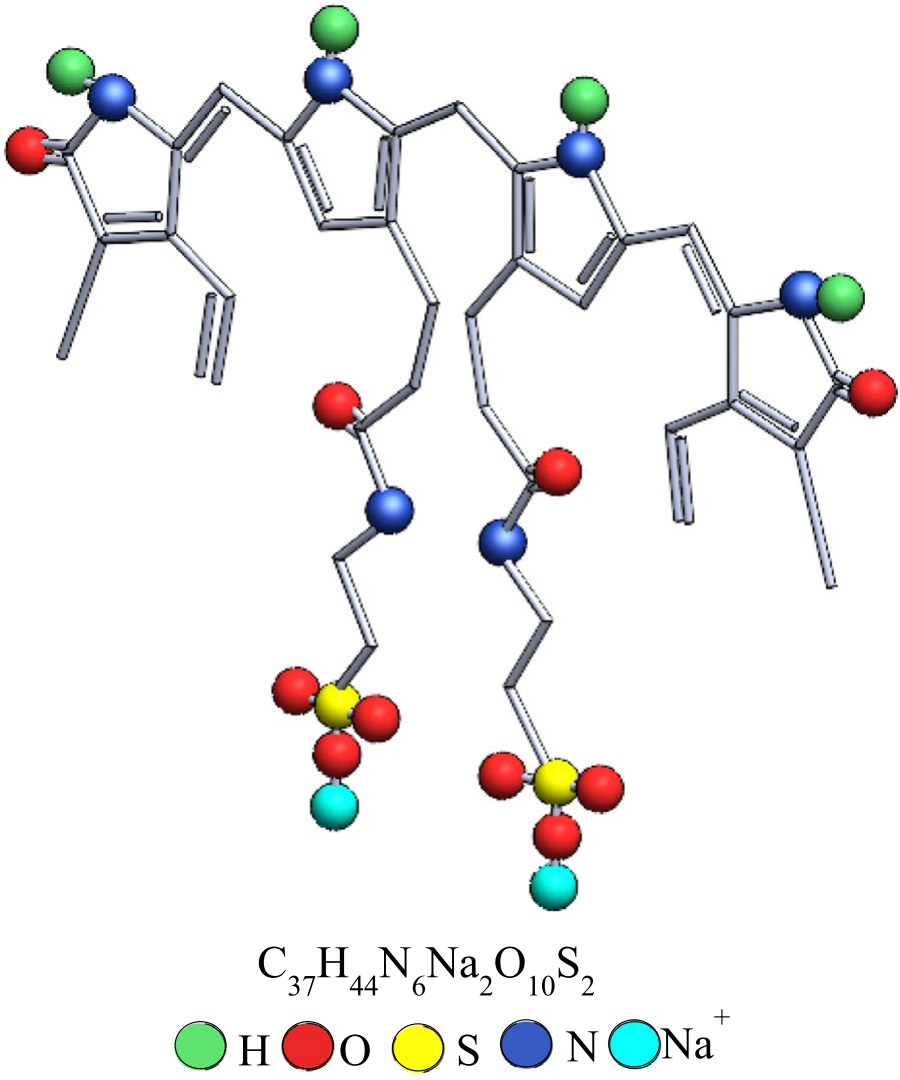
The molecular formula and 3D structure of the direct bilirubin sample

Direct bilirubin powder (bilirubin conjugate, ditaurate, disodium salt – Calbiochem®, Merck Millipore, Inc., USA, Catalog number 201102) is the standard currently used by medical institutions to simulate direct bilirubin. The product’s certificate of analysis was according to Regulation (EC) No. 1907/2006. The molecular formula and 3D structure of the direct bilirubin sample are shown in Fig. 1.

Direct bilirubin powder was mixed with deionized water to form 14 different standard solutions in the concentration range of 0.855–17.1 μmol/L. The weight of direct bilirubin and deionized water was measured using a precision scale and mixed uniformly by stirring at 500 rpm for 5 min with a magnetic stirrer.

### Design for a Mini-LEDs MOD

**Fig. 2:**
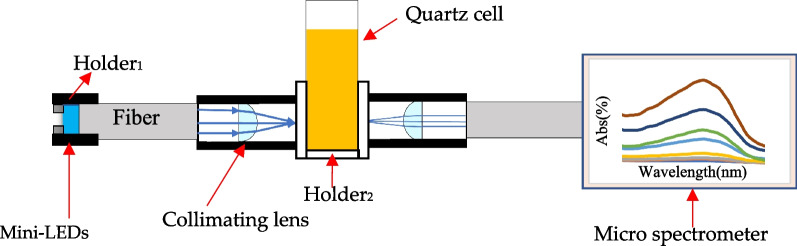
Mini-LEDs MOD optomechanical architecture

The Hitachi U-3900 spectrometer has the disadvantage of being bulky and heavy to carry. This study used mini-LEDs as a light source to design a MOD, which consisted of mini-LEDs, a collimating lens, optical fiber, and a miniature spectrometer, as shown in Fig. 2.

A single mini-LED was fixed and driven on a breadboard. Use tape as a holder_1_ to hold the LEDs and optical fiber when they are centered. When light incident to an optical fiber with a numerical aperture of 0.22, fiber distribution panel of 200 µm, length of 1 m, a diameter of 3.2 mm and focused to a quartz cell that is filled with direct bilirubin through a collimating lens with a back focal length of 10 mm and a numerical aperture of 0.22. The light absorbed through the quartz cell was converted into parallel light by a reverse collimating lens and the incident light on the optical fiber. Finally, different concentrations of direct bilirubin were analyzed by a microspectrometer SE1030-025-FUVN (OtO Photonics, Inc., Hsinchu City, Taiwan). The quartz cell is fixed on the holder_2_, and the collimating lens is fixed on the holder_2_ by threads.

### Experimental Process

**Fig. 3:**
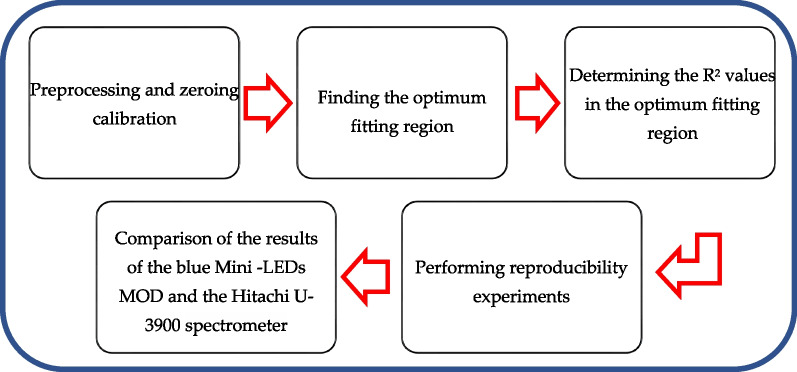
Schematic diagram of the experimental process

The experimental procedure for the detection of direct bilirubin by the MOD developed in this study is shown in Fig. 3. Initially, 14 different concentrations of direct bilirubin standard solutions (0.855 to 17.1 μmol/L) were prepared. The absorbance of air was used as the baseline to zero-calibrate the spectrometer (Hitachi, Ltd., Tokyo, Japan, Model U-3900). After the calibration was complete, the direct bilirubin absorbance values of the standard solutions were measured, and the linear relationship between the absorbance and a standard solution’s concentration was analyzed to find the optimum fitting region. The measured band was then set to the identified optimum fitting region to analyze the relationship between direct bilirubin concentration and absorbance, and the data were analyzed using a binary linear regression; the *R*^2^ value represented the linear relationship between direct bilirubin concentration and the absorption values. To conduct the reproducibility experiments, the developed MOD was rebuilt five times, and the average error of reproducibility was measured for each. Finally, the results of the constructed blue Mini-LEDs MODs and the Hitachi U-3900 spectrometer were compared to verify the developed MOD’s ability to detect direct bilirubin. The MOD experiments will fix the integration time of the microspectrometer and test in the dark room with a room temperature of 25 °C and humidity of 60%.

## Results and Discussion

### Direct Bilirubin Absorbance Spectrum Analysis

**Fig. 4:**
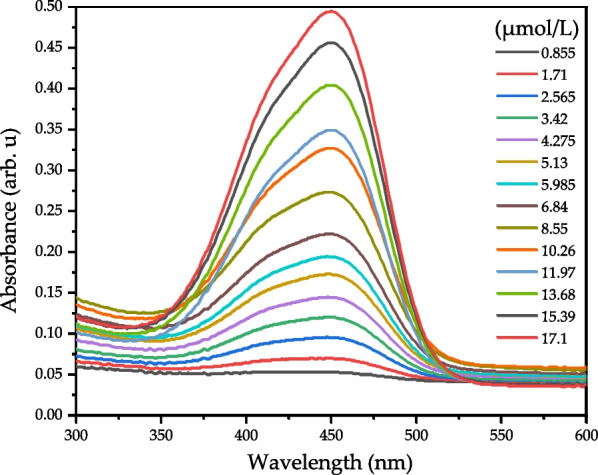
Absorbance spectrum of direct bilirubin measured by the Hitachi U-3900 spectrometer

In this study, direct bilirubin was measured by the Hitachi U-3900 spectrometer first; the absorbance value was measured at a scan rate of 2 nm/s. Fourteen different concentrations of direct bilirubin were prepared, and their absorbance values measured in the concentration range of 0.855–17.1 μmol/L. Figure 4 shows the absorbance spectra of the 14 different concentrations.

**Fig. 5:**
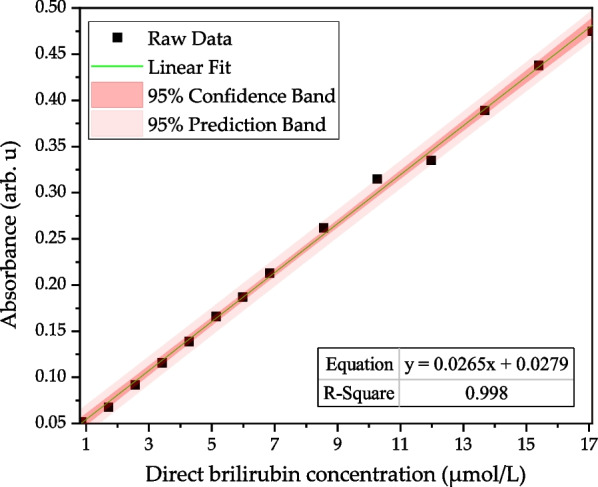
The linear analysis of direct bilirubin (0.855–17.1 μmol/L) by Hitachi U-3900 at 460 nm; the regression equation was *y* = 0.0265*x* + 0.0279, *R*.^2^ of 0.998

The absorbance spectra of direct bilirubin determined that the maximum absorbance wavelength was 448 nm, the linear regression is *y* = 0.0276*x* + 0.0288, and the *R*^2^ reached 0.9978. The optimum fitting wavelength is 460 nm, and the linear regression is *y* = 0.0265*x* + 0.0279 with *R*^2^ value of 0.998 as shown in Fig. 5.

### Detecting Direct Bilirubin Using Blue Mini-LEDs as a Light Source for a MOD

**Fig. 6:**
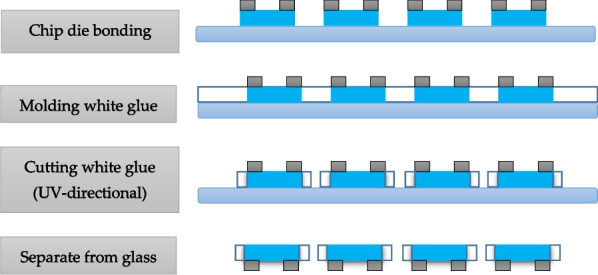
Blue Mini-LEDs packaging production process

This study used Blue Mini-LEDs (Harvatek corporation, Inc., Hsinchu City, Taiwan) as a light source; the chip size length, width, and height were 101.6, 152.4, and 150 µm, respectively. The package size was 800, 800, and 300 µm in length, width, and height respectively. The blue Mini-LEDs package fabrication process is shown in Fig. 6 and includes chip die bonding, molding with white glue, cutting the white glue, and separating it from the glass.

**Fig. 7:**
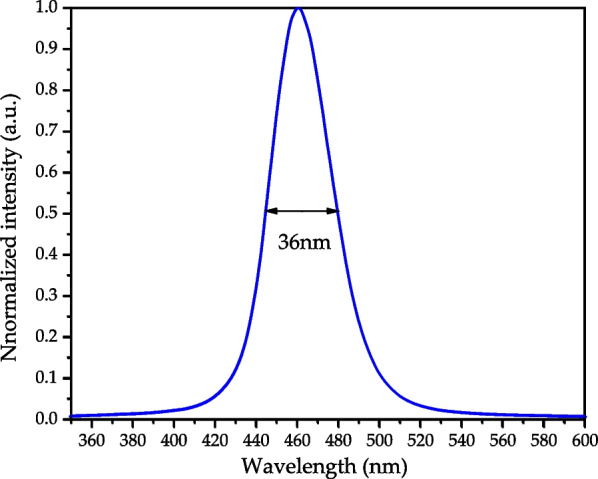
Emission spectra of blue Mini-LEDs

The emission spectrum of the blue Mini-LEDs is shown in Fig. 7. They have a maximum peak at wavelength 462 nm, an optimum fitting range between 390 and 490 nm band light source, and the FWHM of the spectrum is 36 nm.

**Fig. 8:**
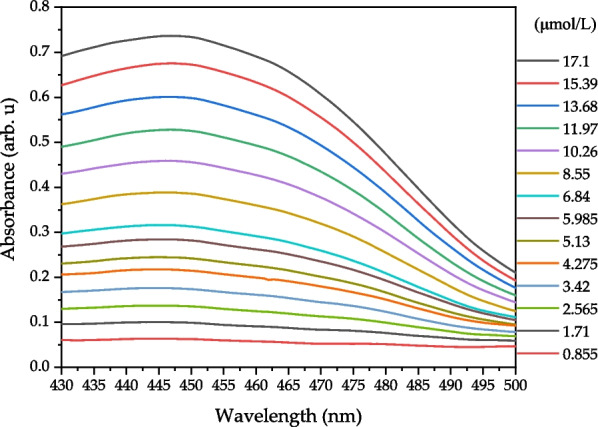
Absorbance spectrum of direct bilirubin at different concentrations measured using the blue Mini-LEDs MOD

The blue Mini-LEDs were driven at 2.8 V and 40 mA. The direct bilirubin was measured in the wavelength range of 430–500 nm as this was the optimum fitting range after analysis. Figure 8 shows the absorbance spectrum of different concentrations (0.855 to 17.1 μmol/L) of direct bilirubin measured by the blue Mini-LEDs MOD.

**Fig. 9:**
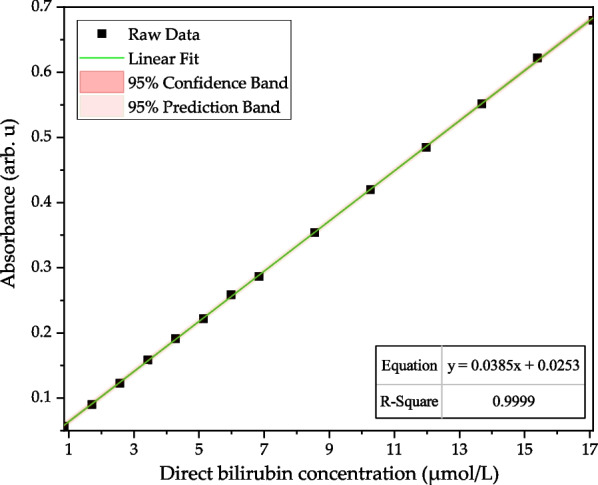
Linear analysis of direct bilirubin (0.855–17.1 μmol/L) by blue Mini-LEDs MOD at 462 nm; the regression equation was *y* = 0.0385*x* + 0.0253, *R*.^2^ of 0.999

The blue Mini-LEDs MOD measurement of direct bilirubin transmission spectrum from concentration 0.855 to 17.1 μmol/L has the best linearity relationship in the band 430 to 500 nm, with the *R*^2^ value reaching above 0.99. Figure 9 shows the optimum fitting wavelength was 462 nm, the linear regression is *y* = 0.0385*x* + 0.0253, the *R*^2^ value is 0.9999, LOD of 0.171 μmol/L, and LOQ of 0.570 μmol/L.

To conduct a reproducibility test, the blue Mini-LEDs MOD was rebuilt five times and each was used to measure three different concentrations of direct bilirubin at 462 nm; the highest concentration was 17.1 μmol/L, the medium concentration was 8.55 μmol/L, and the low concentration was 1.71 μmol/L. The experimental results are shown in Table 1. The standard deviation error (*σ*/√*n*, where *σ* is the standard deviation, and *n* is the number of samples) of the light penetration intensity at wavelength 462 nm of the 17.1, 8.55, and 1.71 μmol/L direct bilirubin was 0.073, 0.068, and 0.140%, respectively, and the average standard deviation error was 0.093%. According to the experimental results, the developed blue Mini-LEDs MOD has excellent reproducibility in the repeated setup and measurement of direct bilirubin.

**Table 1:** The reproducibility measurement of the blue Mini-LEDs MOD

Direct bilirubin concentration (μmol/L)	The absorbance for the first test (arb. u)	The absorbance for a second test (arb. u)	The absorbance for the third test (arb. u)	The absorbance for the fourth test (arb. u)	The absorbance for the fifth test (arb. u)	Standard deviation error (%)
1.71	0.680	0.674	0.674	0.673	0.672	0.140
8.55	0.354	0.352	0.351	0.351	0.350	0.068
17.1	0.090	0.089	0.089	0.087	0.086	0.073

**Fig. 10:**
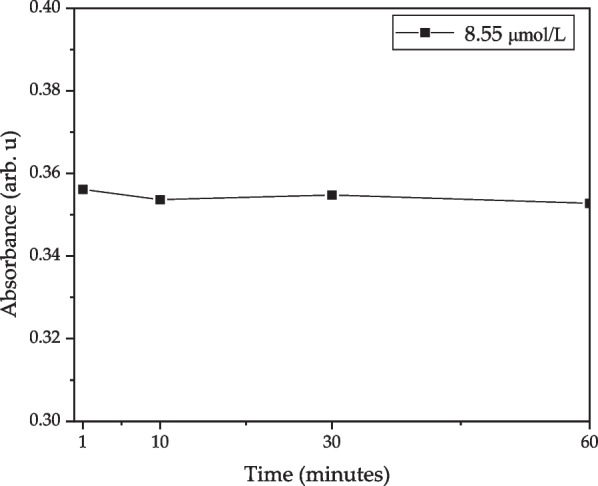
The absorbance for the direct bilirubin after driving the LEDs for 1, 10, 30, and 60 min

The long-term stability test for MOD to detect direct bilirubin of 8.55 μmol/L was tested in a dark room with a room temperature of 25 °C and humidity of 60% as shown in Fig. 10. Besides, we also fix the integration time of the microspectrometer to 1 ms. After driven the LEDs for 1, 10, 30, 60 min, the absorbance for the direct bilirubin concentration of 8.55 μmol/L was 0.35615, 0.35364, 0.35479, 0.35278, respectively, and the standard deviation error was 0.07%.

### Comparing Detection Fitting Between the Blue Mini-LEDs MOD and a Commercial Spectrometer

**Fig. 11:**
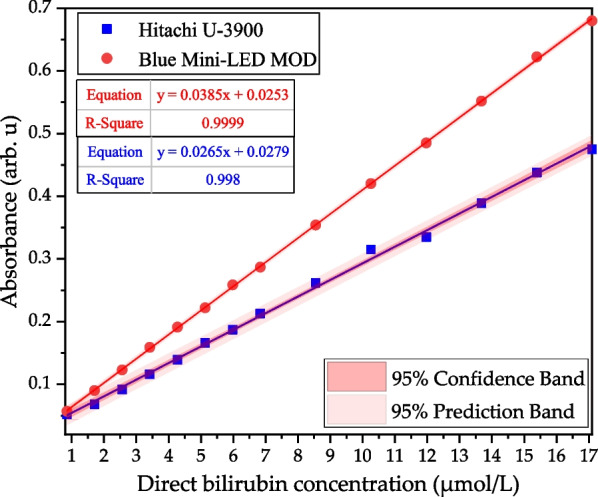
The *R*^2^ value of direct bilirubin absorbance at 462 nm and 460 nm by the blue Mini-LEDs MOD and the Hitachi U-3900 spectrometer

Figure 11 shows the *R*^2^ measured by blue Mini-LEDs MOD and Hitachi U-3900 spectrometer in direct bilirubin (0.855 to 17.1 μmol/L) at the wavelength of 462 nm and 460 nm. The blue Mini-LEDs MOD developed in this study was compared with the Hitachi U-3900 spectrometer measured at a wavelength of 462 nm and 460 nm with different concentrations and absorbance, and the linear regression and *R*^2^ values are *y* = 0.0385*x* + 0.0253, 0.9999 and *y* = 0.0265*x* + 0.0279, 0.998, respectively.

## Conclusions

The experimental data showed that the blue Mini-LEDs MOD developed in this research had a good linear relationship in detecting direct bilirubin when compared with the Hitachi U-3900 spectrometer’s analysis results. Therefore, the developed MOD could simplify the noninvasive quantitative detection of direct bilirubin.

In this study, a noninvasive spectroscopic detection method was proposed to detect direct bilirubin. Blue Mini-LEDs MODs and a Hitachi U-3900 spectrometer were used to analyze the relationship between direct bilirubin at different concentrations and the light absorbance change in the spectrum. The direct bilirubin concentration corresponds to absorbance and absorbance values, and linear regression analysis can provide a tool to achieve a noninvasive quantitative analysis. The experimental results show that the blue Mini-LEDs MOD’s optimal fitting band of direct bilirubin at 14 different concentrations is 462 nm, in the direct bilirubin concentration range from 0.855 to 17.1 μmol/L, the linear regression was *y* = 0.0385*x* + 0.0253, the *R*^2^ is 0.9999, LOD of 0.171 μmol/L, LOQ of 0.570 μmol/L and the average standard deviation error of measurement reproducibility is 0.093%. Comparing the blue Mini-LEDs MOD at 462 nm and the Hitachi U-3900 spectrometer at 460 nm, detect different concentrations of direct bilirubin, the linear regression and *R*^2^ values are *y* = 0.0385*x* + 0.0253, 0.9999 and *y* = 0.0265*x* + 0.0279, 0.998, respectively. Therefore, the blue Mini-LEDs MODs have the advantage of small size and portability which can be used in the future as a noninvasive quantitative detection device for direct bilirubin measurement.

## Data Availability

The data presented in this study are available on request from the all authors.

## Abbreviations

MOD: Miniaturized optomechanical device
Mini-LEDs: Mini-light-emitting diodes
LOD: Limit of detection
LOQ: Limit of quantitation
FWHM: Full width at half maximum
AAP: American Academy of Pediatrics
ODMN: O-desmethylnaproxen
RSD: Relative standard deviation value
BOD: Bilirubin oxidase
MCO: Multicopper oxidase
